# Draft Genome Sequence of *Schaalia odontolytica* Strain ORNL0103, a Basibiont of “*Candidatus* Saccharibacteria” HMT352

**DOI:** 10.1128/MRA.00793-21

**Published:** 2021-11-04

**Authors:** Nicholas A. Podar, Dawn Klingeman, Fabiola Miranda-Sanchez, Floyd E. Dewhirst, Mircea Podar

**Affiliations:** a Oak Ridge High School, Oak Ridge, Tennessee, USA; b Biosciences Division, Oak Ridge National Laboratory, Oak Ridge, Tennessee, USA; c Department of Microbiology, The Forsyth Institute, Cambridge, Massachusetts, USA; d Department of Oral Medicine, Infection, and Immunity, Harvard School of Dental Medicine, Boston, Massachusetts, USA; Indiana University, Bloomington

## Abstract

Here, we report the draft, nearly complete genome sequence of the human oral actinobacterium Schaalia odontolytica strain ORNL0103, which was isolated in association with “*Candidatus* Saccharibacteria” HMT352 strain ORNL0105. The genome was sequenced using a combination of Pacific Biosciences and Illumina platforms and encodes 1,948 proteins and 60 RNAs.

## ANNOUNCEMENT

There are 36 known genera of actinobacteria in the human microbiota, some of which can cause infections (actinomycoses) at different body sites (1). *Actinomyces* is one the most diverse genera, although some of its species have recently been reclassified under a new genus, *Schaalia* (2, 3). In the oral cavity, various actinobacteria have been shown to serve as basibionts (“hosts”) for ectoparasitic saccharibacteria (previously known as candidate division TM7) (4–7). Using labeling of a human saliva sample with a fluorescent antibody against saccharibacteria and single-particle cell sorting on nutrient agar plates, we isolated an actinobacterium (strain ORNL0103) with >99% 16S rRNA identity to *Schaalia* sp. strain HMT180 and Schaalia odontolytica HMT701 (7). ORNL0103 was identified as the basibiont of coisolated “*Candidatus* Saccharibacteria” HMT352 strain ORNL0105 (7) and can also serve as host for other saccharibacteria (HMT952). Streaking of the ORNL0103-ORNL0105 coculture on plates led to identification of colonies of pure ORNL0103 that had lost the saccharibacterium ectobiont.

S. odontolytica ORNL0103 was grown anaerobically (90% N_2_ and 10% CO_2_) in 10-ml aliquots of brain heart infusion (BHI) medium (Difco) for 2 days at 37°C. Genomic DNA (gDNA) was isolated using standard proteinase K digestion followed by phenol-chloroform extraction and ethanol precipitation, as described previously (8). DNA sizing using a Femto Pulse system (Agilent) revealed that >80% of the fragments were between 14 and 20 kbp. For Pacific Biosciences (PacBio) sequencing, a library was prepared directly from unsheared gDNA using the SMRTbell template preparation kit v1.0 (PacBio, Menlo Park, CA) and was sequenced on a PacBio Sequel instrument using a Sequel II 8M single-molecule real-time (SMRT) cell. All data analyses were performed using software defaults unless stated otherwise. The 1.6 million raw reads (*N*_50_, 10.9 kb; maximum, 206 kb) were filtered based on quality values and assembled using HGAP4 in PacBio SMRT Link v7. The polished assembly had 10 contigs and included 61,246 polymerase reads (mean length, 49.7 kb), with 1,045-fold mean coverage. A separate library was prepared using the RhinoSeq high-throughput library preparation kit (SeqOnce Biosciences, Inc.), followed by sequencing (2 × 250-nucleotide reads) on a MiSeq instrument (Illumina, Inc., San Diego, CA). Following quality trimming using Trimmomatic v0.36 in KBase (9), 4.4 million paired-end reads were coassembled with the PacBio reads using hybridSPAdes v3.13.0 (10), resulting in 20 contigs (total length, 2,348,695 bp). The PacBio and hybrid assemblies were annotated using Prokka v1.14.5 (11) and then were coassembled and manually curated in Geneious Prime v2021.1 (12), resulting in 4 contigs with a total length of 2,351,375 bp and a G+C content of 65.6%. Based on the presence of conserved unique genes, as analyzed with CheckM v1.0.18 (13), the genome is 100% complete but not closed.

**FIG 1:**
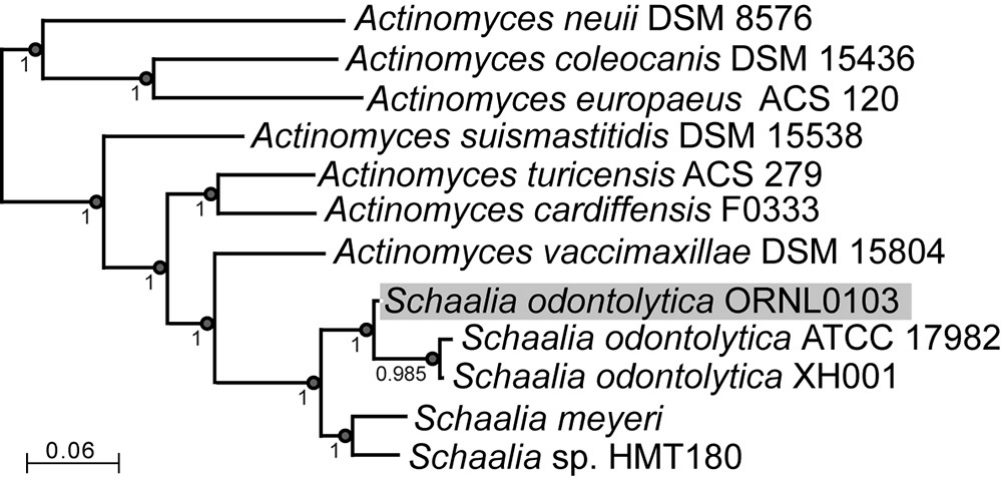
Phylogenetic tree of *Schaalia odontolytica* ORNL0103 and related species and strains based on 49 core, universal bacterial proteins, constructed using KBase Insert Genome Into SpeciesTree v2.2.0, which uses FastTree2 for approximate maximum likelihood phylogeny (https://narrative.kbase.us/#catalog/apps/SpeciesTreeBuilder/insert_set_of_genomes_into_species_tree/release). Numbers at the nodes indicate support values.

To predict and annotate the genes, we used the NCBI Prokaryotic Genome Annotation Pipeline (PGAP) v5.0 (14). The genome encodes 1,948 proteins, 48 tRNAs, 3 rRNA operons (with 23S, 16S, and 5.8S genes), and 3 other small/regulatory RNAs and was compared with other *Schaalia* genomes using FastANI v0.1.2 and SpeciesTreeBuilder v2.2.0 in KBase (9). The genome average nucleotide identity (ANI) between strain ORNL103 and its closest relatives, i.e., S. odontolytica XH001 (6, 15) and S. odontolytica ATCC 17892 (HMT701), is 94.7% and 93.7%, respectively. The ANI of ORNL0103 with *Schaalia* sp. HMT180 strain F0310 is only 84%, and their phylogenetic relationship is shown in Fig. 1. The genome will facilitate study of the diversity of oral actinobacteria and their association with saccharibacteria.

### Data availability.

This whole-genome shotgun project has been deposited in DDBJ/ENA/GenBank under the accession number JAHXSA000000000. The version described in this paper is version JAHXSA000000000. The PacBio and Illumina reads are available under the accession numbers SRR15274828 and SRR15274718, respectively.
